# Utilisation of the 2019 IWGDF diabetic foot infection guidelines to benchmark practice and improve the delivery of care in persons with diabetic foot infections

**DOI:** 10.1186/s13047-021-00448-w

**Published:** 2021-01-28

**Authors:** Matthew Malone, Adriaan Erasmus, Saskia Schwarzer, Namson S. Lau, Mehtab Ahmad, Hugh G. Dickson

**Affiliations:** 1 High Risk Foot Service, Liverpool Hospital, South Western Sydney LHD, Liverpool, Sydney, NSW 2170 Australia; 2 South West Sydney Limb Preservation and Wound Research Academic Unit, South Western Sydney LHD, Liverpool, Sydney, NSW 2170 Australia; 3 Ingham Institute of Applied Medical Research, 1 Campbell Street, Liverpool, NSW 2170 Australia; 4 South West Clinical School, Faculty of Medicine, University of New South Wales, Sydney, Australia; 5 Department of Vascular Surgery, Liverpool Hospital, South Western Sydney LHD, Liverpool, Sydney, Australia

## Abstract

**Aims:**

To utilise the 2019 International Working Group on the Diabetic Foot (IWGDF) - diabetic foot infection (DFI) guidelines as an audit tool for clinical practice in patients with diabetes attending a High-Risk Foot Service.

**Methods:**

Data from 93 consecutive patients were collected over a 19-month period in patients attending a High-Risk Foot Service. The diagnosis and management of each patient in the sample were compared against the 2019 IWGDF DFI guidelines, grouped into four categories: Diagnosis, Microbiology, Treatment of soft tissue infection, and Surgical treatment and osteomyelitis. Deficits in performance were recorded using the recommendations as a benchmark standard.

**Results:**

There were 109 DFI events. Nineteen (63%) of the recommendations were met, 7 (24%) were partially met, and four (13%) recommendations were not met. Fourteen of the sample had no documented requests for full blood counts. Tissue was obtained for culture in 32 (29%) of the sample. No percutaneous bone biopsies were performed. Only 13 (28%) patients had intraoperative bone specimens sent for culture and sensitivities, with no bone specimens sent for histopathology. Modification of antibiotic therapy following available culture results was low, occurring in 12 out of 63 possible occasions (19%). The duration of antibiotic regimens in PEDIS 2 infections and osteomyelitis was greater than that recommended.

**Conclusions:**

Utilising the IWGDF DFI guidelines to benchmark clinical practice is a useful tool to identify gaps in clinical performance or service delivery and may help to improve patient care.

**Supplementary Information:**

The online version contains supplementary material available at 10.1186/s13047-021-00448-w.

## Introduction

Foot infections in persons with diabetes mellitus (DM) are major contributors to increased morbidity, mortality, hospital expenditure and decreased quality of life. The majority of diabetic foot infections (DFIs) occur when the skin envelope is breached, exposing otherwise sterile structures. In persons with DM, this typically occurs in the form of a foot ulceration (DFUs). Reports on the prevalence of infection in DFUs ranges between 9 and 60%, with several risk factors increasing the likelihood of developing an infection. Risk factors for development of infection can include ulcers that penetrate to bone, ulcers of greater durations (> 30 days), recurrent ulcers, ulcers with a traumatic aetiology, and presence of peripheral vascular disease [1].

DFIs are clinically challenging to manage and represent a major causal pathway to lower extremity amputation [1]. The management of DFIs requires a systematic approach akin to a detective trying to solve a case. Attention must be directed toward; diagnosing the pathology, obtaining appropriate specimens for culture and sensitivities to identify pathogens and aid in the selection of antibiotic therapy, rapid decision making regarding surgical intervention or hospitalization, and considering the patient holistically regarding their care requirements [2].

To aid clinicians in providing a systematic, evidence-based clinical approach, the International Working Group on the Diabetic Foot (IWGDF) since 1999 has published evidence-based guidelines on the diagnosis and treatment of foot infection in persons with DM. The IWGDF have further contributed to classifying foot disease states by creating the PEDIS classification. Perfusion, extent, depth, infection, and sensation was designed by the IWGDF for selection of participants for clinical research. This system includes five components: perfusion (PAD), extent (area), depth, infection, and sensation (neuropathy) [3].

The latest iteration from 2019 presents 27 recommendations that cover various aspects of diagnosis of soft tissue and bone infection, including a classification scheme for DFI and its severity [4]. In addition to the clinical utility of the guidance document, the recommendations represent an opportunity for service providers and clinicians to benchmark the care they provide against the defined standard of evidence-based recommendations [5].

In this paper, we use the approach outlined by Standards for Quality Improvement Reporting Excellence (SQUIRE 2.0) [6] to report on data collected on the management of DFIs from a tertiary hospital High-Risk Foot Service. The findings are benchmarked against the IWGDF DFI guidelines/recommendations. The aim of the study was a quality improvement process to understand the current strengths and weaknesses in clinical care in the service provided to individuals with DFI, and to identify areas for enhancement or improvement.

## Methods

### Study design

The data used in this study was from an internal database established by a a major metropolitan tertiary referral hospital High-Risk Foot Service to capture clinical data on Diabetic Foot Infections. The data used was collected over a 19-month period (March 2018 to September 2019), and 93 consecutive eligible patients (109 DFI events) aged over 18 years who presented for multi-disciplinary management of their diabetic foot infection (DFI) were enrolled in the study. Individuals were eligible if they had DM and a new DFI. Individuals could be enrolled on more than one occasion if they presented with multiple DFIs during the study period, but only if the index DFI had completely resolved and treatment was complete. Individuals with foot infections who did not have DM were not eligible for the study.

The High-Risk Foot Service provides a multi-disciplinary team approach, with care provided by podiatrists, endocrinologists, general medicine physicians, infectious diseases physicians and vascular surgeons. Demographic, clinical, laboratory and relevant operation report data were obtained from the electronic medical record, and all extracted data were recorded in a secure Microsoft Excel spreadsheet. Conventional culture data obtained from eMR was reported by a hospital pathology service (Sydney South West Pathology Service). Plates were streaked for isolation onto four quadrants of recommended agars and grown under appropriate atmospheres to isolate clinically relevant organisms (both aerobe and anaerobe) per standardized methods.

The IWGDF DFI guidelines has 27 recommendations which were used as the defined standards for the benchmarking episode. The 27 recommendations fall under four categories: Diagnosis (7), Microbiology (2), Treatment of Soft Tissue Infection (10), and Surgical Treatment and Osteomyelitis (8). Recommendations for antibiotic use in tropical and subtropical regions were not used in the benchmarking process as they were not relevant to the geographical (Sydney, New South Wales) context.

The electronic medical record (eMR) used by the Hospital contains details of all pathology and imaging tests performed on a patient as an inpatient or outpatient, as well as all clinician notations. Antibiotic use was extracted from both the eMR clinical notations and paper medical administration charts used for inpatients. One researcher was tasked with searching the records for information corresponding to the detail required by the standards. Two additional researchers then independently compared actions described in the extracted data grouped in each of the four categories for each patient against the standard of the recommendations. The results were then compared, and a decision made as to whether there was full compliance for the case, partial compliance or no compliance. Disputes were to be resolved with input from a fourth researcher. None of the researchers were blind to the identity of participant data. One senior researcher was involved in data extraction and held managerial responsibility within the High-Risk Foot Service and also undertook a clinical role within the High-Risk Foot Service. The two other researchers involved also undertook a clinical role within the clinic.

### Human research ethics

The study was approved on the 26th November 2020 by the local institutional human research ethics committee (Project ID: 2020/ETH02129) and conducted according to national standards governing clinical research.

### Statistical analysis

All data collected was transcribed into a study specific data collection form, then entered electronically into a secured database. Descriptive data was reported as mean and standard deviation (±). Unpaired students t-tests and one-way analysis of variance (ANOVA) was used for comparisons. Data were analysed using the Statistical Package for Social Sciences Version 25 (SPSS Inc., Chicago, Illinois, USA).

## Results

A total of 93 individuals with 109 DFIs were recruited over the 19-month study period. Sixteen patients included in the dataset had repeat infections of the index infected DFU. The majority of patients had type 2 DM (type 1, 13 [14%]: type 2, 80 [86%]). There were 62 (67%) men and 31 (33%) women. The mean age of all participants was 58.3 ± 12 years. All 109 DFIs were associated with a diabetic foot ulcer (DFU). The location of DFUs and subsequent DFIs were 47 plantar forefoot (43%), 43 digits (40%), 11 midfoot (10%), and 8 calcaneus (7%).

A summary of the clinical results benchmarked against each recommendation is outlined in supplementary data 1. Recommendations are grouped as described earlier. Expanded clinical data is also available for review in supplementary data 2. In addition the analysis of clinical data usinng the statistical tests: unpaired students t-tests and one-way analysis of variance are further reported under each recommendation where conducted.

The IWGDF DFI guidelines list 27 recommendations, and as some are divided into subsections, the total number of responses is 30. The recommendations were met for 19 (63%), partially met in 7 (24%) and were not met in 4 (13%). There were no disputes about interpretation of the data.

### Diagnosis

Of eight possible affirmative responses, five recommendations were met, two were partially met and one was not met. The areas of partial compliance were in obtaining baseline blood tests and a serum biomarker of inflammation. The area not met was in obtaining either a percutaneous or surgical specimen of bone for culture in suspected diabetic foot osteomyelitis (DFO). In 95 of 109 (87%) episodes of DFI, full blood counts were obtained at initial presentation, which included white cell count (mean 10 ± 3 × 10^9/L) and C-reactive protein (mean 46.4 ± 62.8 mg/L). The erythrocyte sedimentation rate was requested and available for 49 of 109 (45%) episodes of DFI (including DFO) (mean 53.5 ± 20 mm/hr). Fourteen (13%) episodes of DFI had no blood tests. Procalcitonin could not be ordered for any DFI as it is not available in our institution’s routine pathology ordering sets.

Forty-six of 109 (42%) episodes of DFI were suspected of having DFO. With regards to the microbiological sampling and histopathology for definitive diagnosis or determining the causative pathogen necessary for targeting treatment, no patient had a percutaneous bone biopsy through healthy skin. In 26 of 46 (57%) episodes of DFI with surgical intervention for management of DFO, 13 of 46 (28%) had intraoperative bone specimens sent for microbiology, culture and sensitivities. No specimens were sent for histopathology.

### Microbiology

Of two recommendations, one was met, and one was partially met. The area of partial compliance was in collecting tissue specimens for culture. The most utilised sampling technique was a wound swab, obtained in 77 (71%) episodes of DFI. Tissue specimens were collected in 32 (29%) episodes of DFI, either by biopsy using a 3 mm biopsy needle or by curettage using a dermal curette.

### Treatment of soft tissue infection

Of ten recommendations, eight were met, and two were partially met. The areas of partial compliance were in modification of empiric antibiotic therapy in view of available culture results, with the preference being to continue broad spectrum antibiotic therapy rather than changing to a narrower spectrum agent. Empiric treatment for DFI was initiated in all 63 (100%) episodes of soft tissue DFI. In 55 (87%) cases there was an initial prescription of oral antibiotic therapy and eight (13%) required parenteral antibiotic therapy. Conventional culture identified 185 isolates from 109 episodes of DFI (Supplementary data 3). The most common isolate was methicillin sensitive *Staphylococcus aureus* (44 [24%], MSSA), which was identified as a mono-microbial infection in 22 DFIs. If methicillin resistant *Staphylococcus aureus* (9 [5%]) isolates are included with MSSA, these organisms account for 53 of 185 (29%) isolates. Streptococci were commonly isolated (*Streptococcus agalactiae* 12 [6.5%]), *Streptococcus milleri* (4 [2%])*, Streptococcus dysgalactiae* (3 [1.6%]). Collectively, aerobic gram-positive cocci accounted for 72 (39%) cultured isolates.

Oral amoxicillin-clavulanate was the most commonly prescribed empiric first-line antibiotic in 30 of 63 (48%) episodes of DFI. This was followed by in rank order: oral clindamycin (8), oral cephalexin (7), intravenous piperacillin and tazobactam (6), oral ciprofloxacin (4), oral dicloxacillin (4 intravenous cephazolin (2), oral trimethoprim/sulphamethoxazole (1), oral doxycycline (1). In 41 (66%) episodes of DFI no alterations to therapy were made despite culture results identifying an organism sensitive to a narrow spectrum agent.

### Surgical treatment and osteomyelitis

Of eight recommendations with 11 possible responses, four were met, two were partially met and three were not met. One of the partially met standards was based on the use of an oral agent, doxycycline, that has limited evidence of efficacy in DFO. The other partially met recommendation was sending proximal bone chips obtained at surgical resection for evidence of residual infection.

The three areas of non-compliance were concerned with the duration of antibiotic therapy. Twenty of 46 (43%) episodes of DFI with DFO were managed with antibiotic therapy and did not undergo surgical intervention. The mean total duration of antibiotic therapy was 14.4 ± 9.1 weeks. The other 26 episodes required surgical intervention for resolution of infection/clinical cure. Of this number, 19 (73%) received a mean duration of (failed) oral antibiotic therapy of 5.8 ± 3.4 weeks prior to requiring surgical intervention. We define failed antibiotic therapy as a failure to achieve infection resolution or clinical cure of the index OM referencing no changes to, or a deterioration of clinical, laboratory and imaging modalities. When combining the total duration of antibiotic therapy to include baseline, surgical intervention and cessation of antibiotic therapy post-surgery, the mean duration of antibiotic therapy in this group was 11.7 ± 6.9 weeks. Seven episodes with DFO required acute admission to hospital for parenteral therapy at baseline due to the severity of infection. In all cases antibiotic therapy was commenced on presentation, and surgical intervention occurred within 7 days of admission. In this group, the mean duration from presentation to cessation of antibiotic therapy was 2 ± 0 weeks.

## Discussion

The management of DFI is clinically challenging and a standardised approach to care requires frequent evaluation and auditing of clinical service delivery. Changing practice to conform to evidence should result in improved outcomes for patients. The reasons for partial and non-compliance are not simply a reflection of lack of knowledge of the evidence but reflect constraints on local resources and the influence of local interpretations of evidence and local policies.

Recommendations 7 and 25 of the IWGDF DFI guidelines specifically relate to obtaining appropriate bone specimens for culture and histopathology. These samples are useful for making or confirming a definitive diagnosis and determining the causative pathogen in DFO. The audit identified that no percutaneous bone biopsies were undertaken, and no bone samples (percutaneous or intraoperative specimens) were sent for histopathology. The most likely explanations for this are: a lack of experience in performing percutaneous biopsy either in an out-patient setting or operating theatre, limited theatre time in a busy tertiary facility, and a failure within local protocols to ensure surgeons obtain intraoperative bone specimens to send for culture and histopathology.

Recommendations 23 and 24 of the IWGDF DFI guidelines provide guidance for the duration and delivery of antibiotic therapy for DFI-associated osteomyelitis. The aim is to ensure that patients do not receive unduly long durations of therapy with the primary aim of reducing selective pressures for antibiotic resistance. With this in mind the recommended duration of antibiotic therapy for DFI-associated osteomyelitis is 6 weeks. The levels of evidence for this recommendation is based mostly on clinical experience [7–10], with only one study having been undertaken on the duration of therapy for DFI-associated osteomyelitis [11]. This study reported that antibiotic therapy for longer than 6 weeks offers no additional benefit. We identified a mean total duration of 14.4 weeks (±9.1 weeks), three times longer than IWGDF recommendations. Until recently, the local practice favoured longer rather than shorter courses of antibiotic therapy. The potential reasons for patients receiving longer durations of therapy most likely reflect the heterogeneity of the cases. Patients may show poor tolerance and adherence to oral antibiotic regimens, refuse surgical debridement or amputation, or a combination of these factors. These suggested explanations were extrapolated from a mixture of sources which included documentation of such reasons in eMR or they were the authors practical experience of working in the facility.

Treatment for DFI-related osteomyelitis in patients who required initial parenteral treatment was also longer than the recommendations. The IWGDF guidelines recommend 5–7 days of parenteral treatment before de-escalating to oral therapy. Our mean total duration was 24 ± 9.8 days. This was not unexpected and reflected established practice regarding outpatient parenteral antimicrobial therapy in the High-Risk Foot Clinic [12]. In January 2019, a study reported that oral antibiotic therapy (usually after at least five to 7 days of intravenous therapy) was non-inferior to intravenous antibiotic therapy when used during the first 6 weeks for osteomyelitis [13]. This study commenced in March 2018, and therefore clinical practice did not reflect the new level of evidence.

The lack of tissue biopsy for culture is difficult to explain. There is evidence indicating that the sensitivity and specificity of tissue specimens for culture results are higher than for swabs [14–16]. All treating clinicians had competency in tissue biopsy, and still favoured swab sampling. This may be partly explained by general contra-indications outlined in local departmental policy (tissue punch biopsies for Podiatrists) such as: location of ulcer near exposed vital structures or bony prominence, uncontrolled anticoagulation therapy, or ulcer size too small to obtain adequate tissue sample. It may also be explained by the patient declining, or time constraints in a busy tertiary facility. However, there might be also be a component of reluctance in pursuing biopsy as the primary sampling method, which may be addressed through regular team debriefings.

Empiric therapy was not often altered, even when available culture results subsequently identified key pathogens amenable to narrowing in the spectrum of antibiotic activity. Only 12 (19%) DFI episodes had alterations to specifically target aerobic Gram-positive cocci, despite these making up a large percentage (39%) of isolates. In all cases, the IWGDF DFI guidelines make recommendations that all mild to moderate (PEDIS 2 and 3) DFIs should be focused to a narrow spectrum of pathogen cover, ideally directed by culture results. In mild to moderate (PEDIS 2 and 3) DFIs this coverage is typically targeted towards the predominant pathogens of infection; aerobic Gram-positive cocci, including *Staphylococcus aureus* and beta-haemolytic Streptococci [17].

In this study, once patients were commenced on empiric regimens, they frequently remained on the same antibiotic for the duration of their therapy. Anecdotal feedback from clinicians responsible for prescribing antibiotics in the High-Risk Foot Service was sought. These highlighted clinicians thought that targeting of regimens were not needed in patients who experienced clinical improvement or resolution of symptoms on empiric regimens, especially since empiric first-line therapies were based on recommendations by local Australian (eTG) guidelines.

Cost and waiting lists restrict the use of some investigations in this study. No patient in the sample had an MRI during the study period. Procalcitonin is not a publicly funded pathology test in Australia and was therefore not utilised.

### Limitations and mitigation

As with any un-blinded audit there is a risk of bias in interpretation of the data. However, data extraction and comparison in this audit is simple, as absences are obvious and not open to dispute. For example, the lack of a pathology result, change in antibiotic or use of hyperbaric oxygen are recorded or not. The use of three researchers improves the accuracy of the findings, and there were no disputes about interpretation of the data. Any absence of expected data was interpreted as a non-performance against the relevant recommendation.

## Conclusion

The IWGDF DFI guidelines present an opportunity for clinicians and service providers to benchmark their current practice against defined expert and evidence-based recommendations. This process has identified several areas of local clinical practice that require change and improvement, commencing with the broader dissemination of the IWGDF guidelines to all relevant staff. The most pressing issue identified with this benchmark process is the lack of access to bone biopsy, either ensuring adequate specimens are obtained intra-operatively and sent for conventional culture or by means of percutaneous biopsy through unaffected skin. The latter in particular identifies a need for service development and upskilling of the workforce, this is a likely reflection of services across Australia.

## Supplementary Information

**Additional file 1: Supplementary data 1**. IWGDF recommendations and the benchmarking process undertaken by three researchers on the diagnosis and management of DFI events. **Supplementary data 2**. Raw data of 93 persons with DFI in 109 DFI events. **Supplementary data 3**. Sunburst diagram of microbiology results from 109 DFI events. A total of 185 microbial isolates were identified, 7 DFI events had no growth on culture.

## Data Availability

Please contact author for data requests.

## Abbreviations

IWGDF: International Working Group on the Diabetic Foot
DFU: Diabetic Foot Ulcer
DFI: Diabetic Foot Infection
DFO: Diabetic Foot Osteomyelitis
SQUIRE: Standards for Quality Improvement Reporting Excellence
DM: Diabetes Mellitus
MSSA: Methicillin sensitive *Staphylococcus aureus*
MRSA: Methicillin resistant *Staphylococcus aureus*
HRFS: High Risk Foot Service
MRI: Magnetic Resonance Imaging
eMR: Electronic Medical Records
PEDIS: Perfusion, Extent, Depth, Infection and Sensation
