# Supplementary material for: Utilisation of the 2019 IWGDF diabetic foot infection guidelines to benchmark practice and improve the delivery of care in persons with diabetic foot infections
Source: J Foot Ankle Res. 2021 Jan 28;14:10. doi: 10.1186/s13047-021-00448-w (PMC7842064; doi:10.1186/s13047-021-00448-w)
Supplement: Supplementary file 1 — Additional file 1: Supplementary data 1. IWGDF recommendations and the benchmarking process undertaken by three researchers on the diagnosis and management of DFI events. Supplementary data 2. Raw data of 93 persons with DFI in 109 DFI events. Supplementary data 3. Sunburst diagram of microbiology results from 109 DFI events. A total of 185 microbial isolates were identified, 7 DFI events had no growth on culture. [file 13047_2021_448_MOESM1_ESM.docx]

Supplementary data 1. IWGDF DFI recommendations and the benchmarking process undertaken by three researchers on the diagnosis and management of DFI events.

|  | **IWGDF recommendation** | **Recommendation met**  **(Yes, No, Partially – traffic light system)** | **Comments** |
| --- | --- | --- | --- |
| Diagnosis | **Recommendation 1:**  a) Diagnose a soft tissue diabetic foot infection clinically, based on the presence of local or systemic signs and symptoms of inflammation. (Strong; Low)  b) Assess the severity of any diabetic foot infection using the Infectious Diseases Society of America/International Working Group on the Diabetic Foot classification scheme. (Strong, Moderate). | **Yes** | All 109 DFI events diagnosed on clinical observations and infection severity were classified using PEDIS. |
|  | **Recommendation 2:**  Consider hospitalizing all persons with diabetes and a severe foot infection, and those with a moderate infection that is complex or associated with key relevant morbidities. (Strong; Low) | **Yes** | Most patients requiring hospitalization had PEDIS 3 and (O) infections, and one patient had PEDIS 4. These patients were considered metabolically unstable, required urgent surgical intervention OR were not amenable to hospital in the home. Nine patients were treated in Hospital in the Home. |
|  | **Recommendation 3:**  In a person with diabetes and a possible foot infection for whom the clinical examination is equivocal or uninterpretable, consider ordering an inflammatory serum biomarker, such as C-reactive protein, erythrocyte sedimentation rate and perhaps procalcitonin, as an adjunctive measure for establishing the diagnosis. (Weak; Low) | **Partially** | Over 3/4 of DFI events had blood tests at baseline which included WCC and CRP.  ESR was requested in under half of DFI events and procalcitonin was not ordered for any DFI event. |
|  | **Recommendation 4:**  As neither electronically measuring foot temperature nor using quantitative microbial analysis has been demonstrated to be useful as a method for diagnosing diabetic foot infection, we suggest not using them. (Weak; Low) | **Yes** | Foot temperatures were not used as part of the infection assessment for DFI in any patient. |
|  | **Recommendation 5:** In a person with diabetes and suspected osteomyelitis of the foot, we recommend using a combination of the probe-to-bone test, the erythrocyte sedimentation rate (or C-reactive protein and/or procalcitonin), and plain X-rays as the initial studies to diagnose osteomyelitis. (Strong; Moderate) | **Partially** | Imaging, PTB test and CRP were performed for all patients with suspected osteomyelitis as well as additional imaging such as CT or leukocyte scintigraphy. MRI in the study institution is not readily available for a timely diagnosis, so CT was the preferred option. |
|  | **Recommendation 6:**  a) In a person with diabetes and suspected osteomyelitis of the foot, if a plain X-ray and clinical and laboratory findings are most compatible with osteomyelitis, we recommend no further imaging of the foot to establish the diagnosis. (Strong; Low).  b) If the diagnosis of osteomyelitis remains in doubt, consider ordering an advanced imaging study, such as magnetic resonance imaging scan, 18F-FDG- positron emission tomography/computed tomography (CT) or leukocyte scintigraphy (with or without CT). (Strong; Moderate) | **Yes**  **Yes** | Standard partially achieved:  1. ESR was only requested in 50% of patients with suspected DFI-associated osteomyelitis (DFO). |
|  | **Recommendation 7:** In a person with diabetes and suspected osteomyelitis of the foot, in whom making a definitive diagnosis or determining the causative pathogen is necessary for selecting treatment, collect a sample of bone (percutaneously or surgically) to culture clinically relevant bone microorganisms and for histopathology (if possible). (Strong; Low) | **No** | Standard not achieved:  1. No percutaneous bone biopsy performed in any DFO event.  2. No histopathology for any DFO event  3. Less than 1/4 of events where surgery was performed had intra-operative specimens sent for microbiologic studies |
| Microbiology | **Recommendation 8:**  a) Collect an appropriate specimen for culture for almost all clinically infected ulcers to determine the causative pathogens. (Strong; Low) AND  b) For a soft tissue diabetic foot infection, obtain a sample for culture by aseptically collecting a tissue specimen (by curettage or biopsy) from the ulcer. (Strong; Moderate) | **Partially** | All DFI events had a culture to identify pathogens of infection and guide antibiotic therapy.  Standards partially achieved:  Low use of tissue biopsy was observed, despite all clinicians having the skills, knowledge and competency to perform soft tissue biopsy or curettage. 71% of cultures were by swab and only 29% by tissue. |
|  | **Recommendation 9:** Do not use molecular microbiology techniques (instead of conventional culture) for the first-line identification of pathogens from samples in a patient with a diabetic foot infection. (Strong; Low) | **Yes** | All cultures were performed using conventional culture-dependant microbiology. |
| Treatment | **Recommendation 10:** Treat a person with a diabetic foot infection with an antibiotic agent that has been shown to be effective in a published randomized controlled trial and is appropriate for the individual patient. Some agents to consider include penicillin’s, cephalosporins, carbapenems, metronidazole (in combination with other antibiotic[s]), clindamycin, linezolid, daptomycin, fluoroquinolones, or vancomycin, but not tigecycline. (Strong; High)  **Recommendation 11:** Select an antibiotic agent for treating a diabetic foot infection based on: the likely or proven causative pathogen(s) and their antibiotic susceptibilities; the clinical severity of the infection; published evidence of efficacy of the agent for diabetic foot infections; risk of adverse events, including collateral damage to the commensal flora; likelihood of drug interactions; agent availability; and, financial costs. (Strong; Moderate). | **Yes**  **Partially** | Antibiotic therapy for DFI events were prescribed in keeping with the electronic Therapeutic Guidelines (eTG) of Australia for diabetic foot infections of the skin and soft tissue and aligned with the clinical severity of infection.  Standard partially achieved:  Targeting of empiric therapy upon return of culture results only occurred in 12 of 63 DFI events, while 41 of 63 started on empiric therapy and remained on this despite available culture results. The predominant antibiotic used in this scenario was oral amoxicillin-clavulanate. |
|  | **Recommendation 12:** Administer antibiotic therapy initially by the parenteral route to any patient with a severe diabetic foot infection. Switch to oral therapy if the patient is clinically improving, has no contraindications to oral therapy and if there is an appropriate oral agent available. (Strong; Low). | **Yes** |  |
|  | **Recommendation 13:** Treat patients with a mild diabetic foot infection, and most with a moderate diabetic foot infection, with oral antibiotic therapy, either at presentation or when clearly improving with initial intravenous therapy. (Weak; Low). | **Yes** |  |
|  | **Recommendation 14:** We suggest not using any currently available topical antimicrobial agent for treating a mild diabetic foot infection. (Weak; Moderate) | **Yes** | No topical agents were used solely for the purpose of treating PEDIS 2 DFI events |
|  | **Recommendation 15:**  a) Administer antibiotic therapy to a patient with a skin or soft tissue diabetic foot infection for a duration of 1 to 2 weeks. (Strong; High)  b) Consider continuing treatment, perhaps for up to 3-4 weeks, if the infection is improving but is extensive, is resolving slower than expected, or if the patient has severe peripheral artery disease. (Weak; Low)  c) If evidence of infection has not resolved after 4 weeks of apparently appropriate therapy, re-evaluate the patient and reconsider the need for further diagnostic studies or alternative treatments. (Strong; Low) | **Partially** | Standards partially achieved:  Average duration of antibiotic therapy for PEDIS 2 was 5 weeks and theoretically these should be 1-2 weeks. PEDIS 3 and 4 required longer duration but this is not unexpected in greater severity of infections. The results maybe a reflection of hospital-based population attending High-Risk Foot clinic as they may have more co-morbidities or increased risk profiles. |
|  | **Recommendation 16:** For patients who have not recently received antibiotic therapy and who reside in a temperate climate area, target empiric antibiotic therapy at just aerobic gram-positive pathogens (beta haemolytic Streptococci and Staphylococcus aureus) in cases of a mild diabetic foot infection. (Strong; Low) | **Yes** |  |
|  | **Recommendation 17:** For patients residing in a tropical/subtropical climate, or who have been treated with antibiotic therapy within a few weeks, have a severely ischemic affected limb, or a moderate or severe infection, we suggest selecting an empiric antibiotic regimen that covers gram-positive pathogens, commonly isolated gram-negative pathogens, and possibly obligate anaerobes in cases of moderate to severe diabetic foot infections. Then, reconsider the antibiotic regimen based on both the clinical response and culture and sensitivity results. (Weak; Low) | **Yes** | Tropical/subtropical climate N/A  Moderate to severe DFI antibiotic coverage described in recommendations 10 and 11. |
|  | **Recommendation 18:** Empiric treatment aimed at Pseudomonas aeruginosa is not usually necessary in temperate climates but consider it if P. aeruginosa has been isolated from cultures of the affected site within the previous few weeks or in tropical/subtropical climates (at least for moderate or severe infection). (Weak; Low) | **Yes** |  |
|  | **Recommendation 19:** Do not treat clinically uninfected foot ulcers with systemic or local antibiotic therapy with the goal of reducing the risk of infection or promoting ulcer healing. (Strong; Low) | **Yes** |  |
| Surgical treatment and osteomyelitis | **Recommendation 20:** Non-surgeons should urgently consult with a surgical specialist in cases of severe infection, or of moderate infection complicated by extensive gangrene, necrotizing infection, signs suggesting deep (below the fascia) abscess or compartment syndrome, or severe lower limb ischemia. (Strong; Low). | **Yes** | The MDT HRFS encompasses the involvement of vascular surgery and so surgeons were frequently consulted for urgent management of DFI. |
|  | **Recommendation 21:**  a) In a patient with diabetes and uncomplicated forefoot osteomyelitis, for whom there is no other indication for surgical treatment, consider treating with antibiotic therapy without surgical resection of bone. (Strong; Moderate)  b) In a patient with probable diabetic foot osteomyelitis with concomitant soft tissue infection, urgently evaluate for the need for surgery as well as intensive post-operative medical and surgical follow-up. (Strong; Moderate) | **Yes**  **Yes** |  |
|  | **Recommendation 22:** Select antibiotic agents for treating diabetic foot osteomyelitis from among those that have demonstrated efficacy for osteomyelitis in clinical studies. (Strong; Low). | **Partially** | Standards partially achieved:  Most (but not all) antibiotic agents used in DFO events have evidence of demonstrated efficacy for OM. However, the use of agents was predominantly by empiric therapy or in medically managed cases targeted through microbiology, culture and sensitivity results from swabs or tissue specimens, not bone. |
|  | **Recommendation 23:**  a) Treat diabetic foot osteomyelitis with antibiotic therapy for no longer than 6 weeks. If the infection does not clinically improve within the first 2-4 weeks, reconsider the need for collecting a bone specimen for culture, undertaking surgical resection, or selecting an alternative antibiotic regimen. (Strong; Moderate)  b) Treat diabetic foot osteomyelitis with antibiotic therapy for just a few days if there is no soft tissue infection and all the infected bone has been surgically removed. (Weak; Low)  **Recommendation 24:** For diabetic foot osteomyelitis cases that initially require parenteral therapy, consider switching to an oral antibiotic regimen that has high bioavailability after perhaps 5-7 days, if the likely or proven pathogens are susceptible to an available oral agent and the patient has no clinical condition precluding oral therapy. (Weak; Moderate) | **No**  **No**  **No** | Standards not achieved:  1. Mean duration of antibiotic therapy was 14.4 weeks. Small percentage of osteomyelitis patients with concomitant skin and soft tissue DFI who had immediate surgical intervention at baseline had the lowest durations of antibiotics.  2. Even following surgical intervention most DFO events received significantly greater durations of therapy then a few days, typically, post-surgical DFO events received weeks of antibiotic therapy.  3. DFO events that were hospitalised and had parenteral therapy at baseline typically remained in parenteral therapy for the duration of hospitalisation which was typically longer then 5-7 days.  4. DFO events requiring parenteral therapy under hospital in the home had average durations of therapy of 24 days (± 9.8). |
|  | **Recommendation 25:**  a) During surgery to resect bone for diabetic foot osteomyelitis, consider obtaining a specimen of bone for culture (and, if possible, histopathology) at the stump of the resected bone to identify if there is residual bone infection. (Weak; Moderate)  b) If an aseptically collected culture specimen obtained during the surgery grows pathogen(s), or if the histology demonstrates osteomyelitis, administer appropriate antibiotic therapy for up to 6 weeks. (Strong; Moderate) | **Partially** | Standard partially achieved:  1. Less than 1/3 of patients undergoing surgery had intraoperative bone specimens sent for microbiology, culture and sensitives. |
|  | **Recommendation 26:** For a diabetic foot infection do not use hyperbaric oxygen therapy or topical oxygen therapy as an adjunctive treatment if the only indication is specifically for treating the infection. (Weak; Low) | **Yes** | Not used in any DFI event |
|  | **Recommendation 27:** To specifically address infection in a diabetic foot ulcer  a) do not use adjunctive granulocyte colony stimulating factor treatment (Weak; Moderate) and,  b) do not routinely use topical antiseptics, silver preparations, honey, bacteriophage therapy or negative pressure wound therapy (with or without instillation). (Weak; Low). | **Yes** | Not used in any DFI event as a sole treatment. Topical antiseptics were frequently used to augment systemic antibiotic therapy as part of localised wound care. |

**Supplementary data 2: Raw data of 93 persons with DFI in 109 DFI events.**

**Diagnosis**

**Recommendation 1:**

All 109 (100%) DFI events were diagnosed based on clinical observations as defined by the International Working Group for the Diabetic Foot (IWGDF) guideline on the diagnosis and treatment of foot infection in persons with diabetes. The classification of infection severity was documented in all 109 (100%) DFI events, and differentiating involvement of skin and soft tissue and/or bone; PEDIS 1 (n= 0, 0%), PEDIS 2 (n= 30, 28%), PEDIS 2(O) (n= 12, 11%), PEDIS 3 (n=41, 38%), PEDIS 3(O) (n= 23, 21%), PEDIS 4 (n=2, 2%), PEDIS 4(O) (n= 1, 1%).

All DFIs were associated with foot ulceration, and the audit of data identified that all 109 DFI events had their foot ulcers graded for size, depth and tissue involvement using classification scheme; Wound, Infection and Foot Ischemia classification - WIfI (REF). DFUs were graded as follows; Grade 0 (n= 0, 0%), Grade 1 (n= 49, 46%), Grade 2 (n = 52, 47%), Grade 3 (n= 8, 7%). The classification scheme also seeks to determine the extent of any arterial disease (Ischemia). In this instance, 90 (83%) DFI events had either appropriate lower extremity arterial tests at the time of presentation or these were requested as part of DFI care; Grade 0 (n= 61, 64%), Grade 1 (n= 8, 8%), Grade 2 (n= 9, 9%), Grade 3 (n= 10, 11%). Lower extremity arterial tests were not available or were not requested in 19 (17%) individuals.

**Recommendation 2:**

The MDT HRFS service has established pathways in place for urgent reviews of patients presenting with a new DFI and concomitant systemic inflammatory response syndrome (SIRS), or otherwise determined to require hospitalization. In patients presenting with a new index DFI, 15 of 109 (14%) DFI events required immediate admission through the foot service for in-patient care, either for immediate surgical intervention and/or parenteral therapy (PEDIS 3 = 6, PEDIS 3(O)= 7, PEDIS 4 = 1). A further nine (8%) patients were deemed as requiring parenteral therapy, however, hospital avoidance was achieved through the use of Hospital in the Home (HITH) services. (PEDIS 2(O)= 3, PEDIS 3= 3, PEDIS 3(O)= 3).

**Recommendation 3:**

95 of 109 (87%) DFI events had reportable full blood counts at initial presentation, which included white cell count (WCC) (mean WCC = 10 x10^9/L, ± 3) and the inflammatory marker; C-reactive protein (CRP) (mean CRP = 46.4 mg/L ± 62.8). The inflammatory marker; erythrocyte sedimentation rate (ESR) was only requested and available for 49 of 109 (45%) DFI events (mean ESR = 53.5 mm/hr, ± 20). Fourteen (13%) DFI events had no blood tests. The inflammatory marker Procalcitonin was not ordered for any DFI event.

**Recommendation 4:**

Foot temperatures were not used as part of the infection assessment for DFI in any patient in this cohort. Similarly, neither quantitative or semi-quantitative conventional culture were used for diagnostic purposes to define the presence or absence of infection in any patient in this cohort.

**Recommendations 5-7:**

46 of 109 (42%) DFI events were suspected of having osteomyelitis (DFO – Diabetic Foot Osteomyelitis). 20 of 46 (43%) suspected DFO cases were located at the digits, 18 (39%) located at the plantar forefoot, 4 (9%) at the midfoot and 4 (9%) at the calcaneus. Of the 46 DFI events with suspected DFO, 38 (83%) events were suspected with DFO at baseline, and a further eight (17%) DFI events were suspected with DFO at a later stage of their management (3.8 weeks from baseline, ± 1.8 weeks). Of the 38 DFI events suspected with DFO at baseline, imaging, the probe to bone test and CRP were performed in all cases; plain X-ray = 19 (50%), plain X-ray and computed tomography (CT) = 13 (34%), CT alone = 3 (8%), plain X-ray and leukocyte scintigraphy = 3 (8%), negative probe to bone = 13 (34%), positive probe to bone = 25 (66%), CRP = 41.8 mg/L (±39.6), ESR = 67.8 mm/hr, (± 33). Only 19 of 38 (50%) suspected DFO cases had ESR. In eight DFI events, DFO was suspected in the later stages of management and diagnosis was performed using a combination of imaging and the probe to bone test in all cases; plain X-ray = 6 (75%), plain X-ray and computed tomography (CT) = 2 (25%), negative probe to bone = 5 (63%), positive probe to bone = 3 (37%). Inflammatory markers were undertaken and available in six of eight suspected DFO events (75%); erythrocyte sedimentation rate (ESR) (mean ESR = 79.5 mm/hr, ± 32.3) and C-reactive protein (CRP) (mean CRP = 61 mg/L ± 41).

There was no magnetic resonance imaging or 18F-FDG- positron emission tomography/computed tomography (CT) scans used for any patient with suspected DFO. With regards to the microbiological sampling and histopathology for definitive diagnosis or determining the causative pathogen necessary for selecting treatment, no patient (0 of 46, 0%) had a percutaneous bone biopsy through healthy skin. In 26 of 46 (57%) DFO events who underwent surgical intervention for management of DFO, 13 of 46 (28%) had intraoperative bone specimens sent for microbiology, culture and sensitives, and no specimens were sent for histopathology. The most commonly reported isolates are noted in figure 3.

**Microbiology**

**Recommendation 8:**

All 109 DFI events had reportable wound cultures for conventional microbiology, culture and sensitivities. The most utilised sampling technique was a wound swab obtained in 77 (71%) DFI events. Tissue specimens were collected in 32 (29%) DFI events, either by biopsy using a 3mm biopsy needle or by curettage using a dermal curette.

**Recommendation 9:**

Molecular microbiology (DNA sequencing) was not used in any patient (n = 0 / 100, 0%) for the purpose of diagnosis of DFI.

**Treatment of skin and soft tissue infections**

**Recommendations 10 and 11:**

For the analysis of skin and soft tissue DFIs only, 46 DFO events were removed from analysis (Data reviewed under recommendation 22) leaving 63 (58%) skin and soft tissue DFIs. Empiric treatment for DFI was initiated in all 63 (100%) events. 55 (87%) DFI events were commenced on oral antibiotic therapy and eight (13%) required parenteral therapy. Conventional culture identified 185 isolates from 109 DFI events (Figure 2). The most common isolate was methicillin sensitive *Staphylococcus aureus* (MSSA = 44 of 185, 24%), identified as a monomicrobial infection in 22 DFI events. If methicillin resistant *Staphylococcus aureus* (MRSA = 9, 5%) isolates are included with MSSA (MSSA + MRSA = 53 of 185 isolates), *Staphylococcus aureus* represents a major pathogen of infection. Further, Streptococci were commonly isolated (*Streptococcus agalactiae* = 12 (6.5%), *Streptococcus milleri* = 4 (2%)*, Streptococcus dysgalactiae* = 3, 1.6%). Collectively, aerobic gram-positive cocci accounted for 39% (n= 72 of 185) of cultured isolates.

Oral amoxicillin-clavulanate was the most commonly prescribed empiric, first line antimicrobial in 30 of 63 (48%) DFI events. This was followed by in rank order; oral clindamycin (n= 8 of 63, 13%), oral cephalexin (n= 7 of 63, 11%), intravenous piperacillin and tazobactam (n= 6 of 63, 10%), oral ciprofloxacin (n= 4 of 63, 6%), oral dicloxacillin (n= 4 of 63, 5%) intravenous cephazolin (n= 2 of 63, 3%), oral trimethoprim/sulphamethoxazole (n = 1 of 63, 2%), oral doxycycline (n = 1 of 63, 2%). In 41 (66%) DFI events who commenced on empiric antibiotic therapy, no alterations to therapy were made despite returning culture results.

12 (19%) DFI events had alterations to antibiotic regimens that were a targeted approach towards aerobic Gram-positive cocci, or gram-positive and gram-negative bacteria following microbiology, culture and sensitivities; oral amoxicillin-clavulanate (n= 3, 25%), oral dicloxacillin (n= 2 of 12, 17%), oral flucloxacillin (n= 2 of 12, 17%), oral trimethoprim/sulphamethoxazole (n = 2 of 12, 17%), oral clindamycin (n=1 of 12, 8%), oral ciprofloxacin (n = 1 of 12, 8%), Oral linezolid (n= 1 of 12, 8%).

A further 10 (16%) DFI events who commenced on oral antibiotic therapy experienced deterioration of infective symptoms and required escalation to parenteral therapy which were based on available microbiology, culture and sensitivity results; piperacillin and tazobactam (n= 5 of 10, 50%), cephazolin (n= 2 of 10, 20%), vancomycin (n= 2 of 10, 20%), teicoplanin (n= 1 of 10, 10%).

**Recommendation 12:**

Only two of 63 (3%) DFI events presented with PEDIS 4 infections during the study period. One DFI event had a hospital admission for parenteral therapy and surgical intervention and was later discharged on oral antibiotic therapy. One DFI event had oral antibiotic therapy after refusing parenteral therapy and hospital admission and experienced infection resolution with close follow up in the MDT-HRFS.

**Recommendation 13:**

All PEDIS 2 DFI events (n=30 of 63, 48%) and 24 (38%) PEDIS 3 DFI events were treated at baseline with empiric oral antibiotic therapy. Eight (13%) PEDIS 3 DFI events were treated with empiric parenteral therapy. In eight DFI events initiated on parenteral therapy, six (75%) were deescalated to continue on oral therapy and two (25%) DFI events required only parenteral.

**Recommendation 14:**

No mild (PEDIS 2) DFI events were treated solely with a topical antimicrobial for the purpose of infection management.

**Recommendation 15:**

The average duration of antibiotic therapy for skin and soft tissue DFI was six weeks (± 5.7 weeks). There was individual DFI event heterogeneity between PEDIS infection grades, the patterns of infection resolution or failure, and thus, in the duration of total therapy, but this was not statistically significant (ANOVA, *p*= .47). The categories of antibiotic durations (in weeks) provided in the management of DFI are as follows; 21 of 63 (33%) DFI events had antibiotic durations of 1-2 weeks, 15 of 63 (24%) DFI events had antibiotic durations of between 3-4weeks, 14 of 63 (22%) DFI events had antibiotic durations of ≥4 weeks ≤10 weeks and 13 of 63 (21%) DFI events had antibiotic durations of ≥11 weeks. The mean duration of antibiotic therapy by PEDIS infection grade was; PEDIS 2 DFI events was five weeks (± 4.5 weeks), PEDIS 3 was 6.7 weeks (± 6.3 weeks) and PEDIS 4 was eight weeks (± 5.6 weeks). Antibiotic duration was further assessed for differences against the absence or presence and severity of peripheral arterial disease using WIfI. There were no significant differences detected in the mean durations (weeks) of antibiotic therapy (ANOVA, *p*= 1.2); Grade 0 = 8.1 weeks (±8.2 weeks), Grade 1 = 11.1 weeks (±8.8 weeks), Grade 2 = 10.1 weeks (±8.6 weeks), Grade 3 = 11.8 weeks (±7.2 weeks).

11 of 63 (17%) DFI events required escalation of oral antibiotic therapy to parenteral delivery for either deteriorating clinical symptoms or non-response to initial therapy. 15 of 63 (24%) DFI events affecting only the skin and soft tissue required surgical intervention following a period of failed medical management with antibiotics. The most common procedures were either incision and drainage with debridement of soft tissue (n= 13, 21%), or amputation and wide excision to remove all infected tissue and adjacent structures (n = 2, 3%).

**Recommendation 16:**

The pattern of prescribing for PEDIS 2 DFIs were generally targeting or providing primary coverage towards aerobic Gram-positive cocci and were in keeping with Australian therapeutic guidelines (eTG); Oral amoxicillin-clavulanate (n= 14 of 30, 47%), oral cephalexin (n= 5 of 30, 17%), oral clindamycin (n= 3 of 30, 10%), oral dicloxacillin (n= 2 of 30, 7%), oral flucloxacillin (n= 1 of 30, 3%).

**Recommendation 17:**

5 of 30 PEDIS 2 DFI events were in patients either recently treated with other oral antibiotics for the index DFI, or had an available microbiology, culture and sensitivity history which supported the clinical use of a specific antibiotic regimen. These included; oral doxycycline (n= 1 of 30, 3%), oral trimethoprim/sulphamethoxazole (n = 1, 2%), oral ciprofloxacin (n=2 of 30, 7%), oral ciprofloxacin plus clindamycin (n= 1 of 30, 3%). The selection of an empiric antibiotic regimen that covers gram-positive pathogens, commonly isolated gram-negative pathogens, and possibly obligate anaerobes in cases of moderate to severe diabetic foot infections have been described in recommendations 10 and 11.

**Recommendation 18:**

No DFI events were treated empirically for the purpose of targeting Pseudomonas aeruginosa.

**Recommendation 19:**

The study excluded non-infected DFUs and therefore we cannot report against this metric.

**Surgical treatment and osteomyelitis**

**Recommendation 20:**

46 of 109 (42%) DFI events were diagnosed with osteomyelitis (DFO – Diabetic Foot Osteomyelitis). 26 of 46 (57%) skin and soft tissue DFI events with concomitant DFO underwent surgical intervention. This included; Incision and drainage with local debridement/resection of soft tissue and bone or minor amputation of a digit/s below the ankle (n= 25, 96%), or major amputation (n = 1 of 26, 4%) defined as being above the ankle. In total, there were 34 of 109 (29%) events that required surgical intervention in this study. Eight (24%) surgical interventions were for skin and soft tissue DFI, and 26 (76%) were for DFI with DFO.

**Recommendation 21:**

20 of 46 (43%) DFO events were medically managed (non-surgical). For the purpose of this recommendation, “uncomplicated forefoot DFO” was defined in this study as the absence of cellulitis or complex skin and soft tissue involvement of deep structures. Based on this, 10 of 20 (50%) medically managed uncomplicated forefoot DFO events received only oral antibiotic therapy for the duration of their care. An additional 4 of 20 (20%) uncomplicated DFO events were initiated on oral antibiotic therapy at baseline of the index infection, but experienced deterioration/worsening of infective symptoms of the skin and soft tissue and required escalation to parenteral therapy. All cases were subsequently deescalated back to oral antibiotic therapy on improvement of symptoms. Six of 20 (30%) medically managed DFO events were classified as having skin and soft tissue involvement at baseline of the index infection and required initial parenteral therapy due to this complicating factor. All patients were deescalated to oral antibiotic therapy on improvement of symptoms.

46 of 109 (42%) DFI events were diagnosed with osteomyelitis (DFO – Diabetic Foot Osteomyelitis). 26 of 46 (57%) skin and soft tissue DFI events with concomitant DFO underwent surgical intervention. This included; Incision and drainage with local debridement/resection of soft tissue and bone or minor amputation of a digit/s below the ankle (n= 25, 96%), or major amputation (n = 1 of 26, 4%) defined as being above the ankle. In total, there were 34 of 109 (29%) events that required surgical intervention in this study. Eight (24%) surgical interventions were for skin and soft tissue DFI and 26 (76%) for DFI with DFO.

**Recommendation 22:**

20 of 46 (43%) DFO events were medically managed with antibiotic therapy and did not undergo surgical intervention. The following antibiotic regimens were utilised in medically managed uncomplicated forefoot DFO; oral amoxicillin-clavulanate (n= 9 of 15, 60%), oral clindamycin (n=1 of 15, 7%), oral flucloxacillin (n=1 of 15, 7%), oral cephalexin (n=1 of 15, 7%), ciprofloxacin and clindamycin (n=3 of 15, 19%). Six of 20 skin and soft tissue DFI events with concomitant DFO (PEDIS 2(O)= 3, PEDIS 3(O)= 3) required parenteral therapy at baseline, of which the antibiotics used were; piperacillin and tazobactam (n=2 of 5, 40%), cephazolin (n=1 of 5, 20%), cephazolin and oral metronidazole (n=1 of 5, 20%), vancomycin (n=1 of 5, 20%). Following clinical improvement of skin and soft symptoms, all patients were deescalated to oral antibiotic therapy that were culture and sensitivity driven; oral flucloxacillin (n= 2 of 5, 40%). oral trimethoprim/sulphamethoxazole (n=1 of 5, 20%), ciprofloxacin and clindamycin (n=2 of 5, 40%).

26 of 46 (57%) DFO events required surgical intervention for resolution of infection. In 19 of 26 (73%) DFO events, a period (5.8 weeks, ± 3.4 weeks) of failed medical management via antibiotic therapy occurred prior to surgical intervention. The antibiotic regimens used were; oral amoxicillin-clavulanate (n= 10 of 19, 53%), oral clindamycin (n=3 of 19, 16%), oral ciprofloxacin and clindamycin (n=2 of 19, 11%), oral cephalexin (n=1 of 19, 5%), oral trimethoprim/sulphamethoxazole (n=1 of 19, 5%), oral trimethoprim/sulphamethoxazole and metronidazole (n=1 of 19, 5%). 7 of 26 (27%) DFO events required parenteral therapy at baseline for concomitant skin and soft tissue DFI which were commenced prior to surgical intervention which occurred <7 days from the day of admission in all cases. The parenteral antibiotic regimens used were; piperacillin and tazobactam (n=5 of 7, 72%), cephazolin and oral metronidazole (n=1 of 7, 14%), vancomycin (n=1 of 7, 14%).

**Recommendation 23 and 24**

20 of 46 (43%) DFO events were medically managed with antibiotic therapy and did not undergo surgical intervention. The mean total duration of antibiotic therapy was 14.4 weeks (±9.1 weeks). 26 of 46 (73%) DFO events required surgical intervention for resolution of infection. Of this number, 19 of 26 (73%) received a mean duration of (failed) oral antibiotic therapy of 5.8 weeks (± 3.4 weeks) prior to surgical intervention. When combining the total duration of antibiotic therapy to include baseline, surgical intervention and cessation of antibiotic therapy post-surgery, the mean duration of antibiotic therapy in this group was 11.7 weeks (± 6.9 weeks). 7 of 26 (27%) DFO events required admission to hospital for parenteral therapy at baseline for concomitant skin and soft tissue DFI. Antibiotic therapy was commenced on presentation, and these events underwent surgical intervention which occurred within <7 days from the day of admission in all cases. In this group, the total duration of therapy from presentation to surgery, to cessation of antibiotic therapy, was 2 weeks (± 0 weeks).

The total durations for medical management via antibiotic therapy alone (n= 20) and failed medical management with surgical intervention (n= 26) were not significantly different (antibiotic alone = 14.4 weeks versus antibiotic-surgery-antibiotic = 11.9 weeks, *p* = 0.2). In 7 of 26 (27%) DFO events requiring admission for parenteral therapy and immediate surgery, the mean total durations of antibiotic therapy were significantly less than events being managed by antibiotic therapy (oral or parenteral) alone or failed medical management (antibiotic therapy) and surgery (ANOVA, *p* = .002). Six DFO events were classified as hospital avoidance and this was achieved through the use of Hospital in the Home (HITH) services for delivery of parenteral antibiotic therapy (PEDIS 2(O)= 3, PEDIS 3= 3, PEDIS 3(O)= 3). The average total duration of parenteral antibiotic therapy was 24 days (±9.8 days).

**Recommendation 25:**

In 26 of 46 (43%) DFO events who underwent surgical intervention, 13 (28%) had intraoperative bone specimens sent for microbiology, culture and sensitives. The most commonly reported isolates are noted in figure 3.

**Recommendation 26 and 27:**

No patients (n = 0 / 100, 0%) during the study period were referred for hyperbaric oxygen therapy or had adjunctive granulocyte colony stimulating factor treatment. Additionally, no patients with DFI received topical antiseptics, silver preparations, honey, bacteriophage therapy or negative pressure wound therapy (with or without instillation) as a primary treatment for DFI, in place of systemic antibiotics.

**Supplementary data 3:** Sunburst diagram of microbiology results from 109 DFI events. A total of 185 microbial isolates were identified, 7 DFI events had no growth on culture.

**
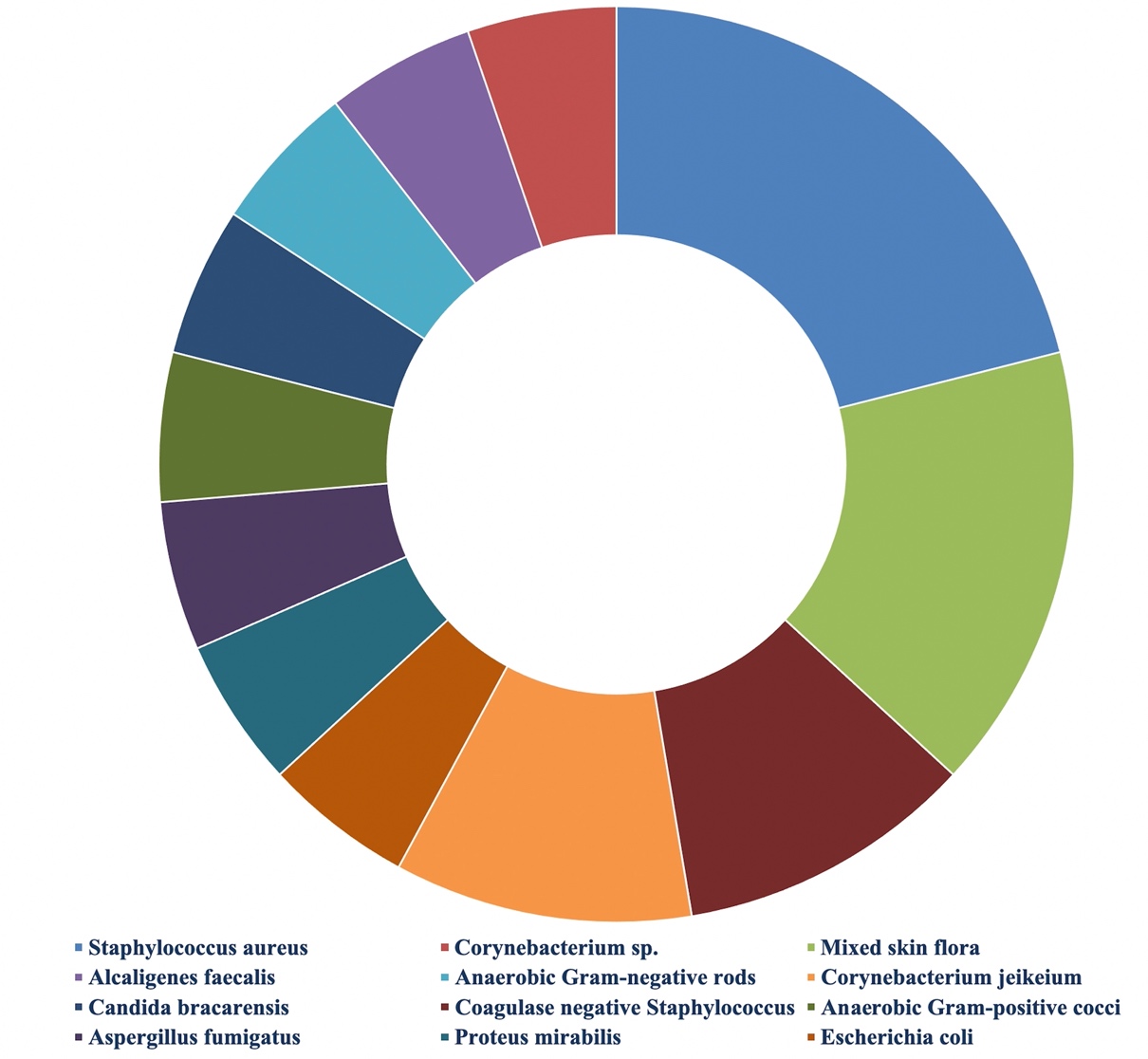
**
